# A pink mouse reports the switch from red to green fluorescence upon Cre-mediated recombination

**DOI:** 10.1186/1756-0500-5-296

**Published:** 2012-06-14

**Authors:** Heiner Hartwich, Somisetty V Satheesh, Hans Gerd Nothwang

**Affiliations:** 1 Department of Neurogenetics, Institute of Biology and Environmental Sciences, Carl von Ossietzky University, Carl-von-Ossietzky-Straße 9-11, 26129, Oldenburg, Germany; 2 Research Center Neurosensory Science, Carl von Ossietzky University Oldenburg, Carl-von-Ossietzky-Straße 9-11, 26129, Oldenburg, Germany

## Abstract

**Background:**

Targeted genetic modification in the mouse becomes increasingly important in biomedical and basic science. This goal is most often achieved by use of the Cre/loxP system and numerous Cre-driver mouse lines are currently generated. Their initial characterization requires reporter mouse lines to study the *in vivo* spatiotemporal activity of Cre.

**Findings:**

Here, we report a dual fluorescence reporter mouse line, which switches expression from the red fluorescent protein mCherry to eGFP after Cre-mediated recombination. Both fluorescent proteins are expressed from the ubiquitously active and strong CAGGS promoter. Among the founders, we noticed a pink mouse line, expressing high levels of the red fluorescent protein mCherry throughout the entire body. Presence of mCherry in the living animal as well as in almost all organs was clearly visible without optical equipment. Upon Cre-activity, mCherry expression was switched to eGFP, demonstrating functionality of this reporter mouse line.

**Conclusions:**

The pink mouse presented here is an attractive novel reporter line for fluorescence-based monitoring of Cre-activity. The high expression of mCherry, which is visible to the naked eye, facilitates breeding and crossing, as no genotyping is required to identify mice carrying the reporter allele. The presence of two fluorescent proteins allows in vivo monitoring of recombined and non-recombined cells. Finally, the pink mouse is an eye-catching animal model to demonstrate the power of transgenic techniques in teaching courses.

## Findings

### Background

Spatiotemporally restricted genetic modification in the mouse is becoming an indispensable tool in biological research [1]. Applications include analysis of essential genes, whose constitutive ablation results in embryonic/perinatal lethality, discrimination between primary and secondary effects, or mapping cell connectivity and cell fate [2-4]. The importance of targeted genetic modification is underlined by a recent global initiative to generate conditional alleles for all murine genes [5-7]. Most targeted genetic modifications make use of the Cre/loxP system. In this approach, a Cre-driver mouse line, expressing Cre recombinase of the phage P1 under a tissue or cell-type specific promoter, is crossed to mice with a loxP flanked (“floxed”) allele, which is recognized by Cre [8].

Several Cre-driver lines have been generated and each of them requires initial characterization of the spatial and temporal Cre expression pattern [9]. A popular reporter line expresses LacZ from the ROSA26 locus upon Cre-mediated recombination [10], but its analysis is limited to fixed tissue. For *in vivo* observation of Cre-mediated recombination, several fluorescent reporter lines have been generated [11-15]. However, only few dual-reporter lines are available with different labels of recombined and non-recombined cells. Some of them combine a fluorescent protein with an enzyme (alkaline phosphatase or LacZ) [16-18], others combine two different fluorescent proteins [19,20]. Here we present a novel Cre-reporter line with high and ubiquitous expression of two fluorescent proteins and which is easily recognized by its pink skin color.

## Materials and methods

### Transfection of HEK-293 cells

The plasmid pCAGGS_cherry_Intron_GFP was transiently transfected in HEK-293 cells with TurboFect (MBI Fermentas, St. Leon-Rot, Germany) according to the manufacturer´s instructions.

### Generation and genotyping of transgenic mice

**Figure 1:**
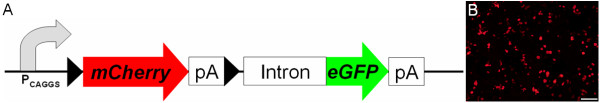
**The construct and its verification. A**) The construct consists of a *loxP* flanked mCherry cassette, which is driven by the chicken β-actin promotor with CMV enhancer, followed by an intron-*eGFP* cassette, which will only be expressed after Cre-mediated recombination. Black triangles illustrate the *loxP* sites. **B**) Expression of mCherry was validated in HEK-293 cells after transient transfection. Scale bar is 100 μm.

A 5.3 kb fragment containing the promoter, the open reading frames for mCherry and eGFP, and the polyadenlation signal were released from the plasmid pCAGGS_cherry_Intron_GFP by restriction with *Mss*I and *Ecl*136II (MBI Fermentas). The plasmid contains a CAGGS promoter, followed by a *loxP* flanked *mcherry-pA* cassette and a β-actin intron followed by the sequence of *egfp-pA* (Figure 1A) The entire transgenic construct is flanked by FRT sites. After gel electrophoresis in low melting agarose, the fragment was purified using agarase according to the manufacturer´s instructions (MBI Fermentas). The final construct was dissolved in microinjection buffer (5 mM Tris, 0.1 mM EDTA, pH 7.6). Pronucleus injection was carried out at the Transgenic Core Facility of the Max-Planck Institute of Molecular Cell Biology and Genetics at Dresden. Genotyping of the transgenic mice was performed using the primers 5´-CGTAATGCAGAAGAAGACCATGG-3´ and 5´-CCTGCTATTGTCTTCCCAATCC-3´. The Cre-driver line *Egr2*^*Cre/+*^ has been described previously [21].

All animals used for these experiments were maintained on C57BL/6 J (Harlan Winkelmann, Borchen, Germany) and C57BL/6 N (Charles River, Sulzfeld, Germany) mixed genetic backgrounds. All protocols adhered to the German Animal Protection law and were approved by the local animal care and use committee (LAVES, Oldenburg). Protocols also followed the NIH guide for the care and use of laboratory animals.

### Determination of transgene copy number

Transgene copy number was determined by semi-quantitative PCR, using the primers 5´-AAACATAACTTCGTATAATGTATGCTATACG-3´ and 5´-CGTAATGCAGAAGAAGACCATGG-3´ with the following conditions: denaturation for 5 min at 95 °C and 30 sec at 95 °C, annealing for 30 sec at 53 °C, 30 sec, synthesis for 30 sec at 72 °C and 5 min at 72 °C, 30 cycles with selfmade Taq-Polymerase. As a standard, we used the transgenic construct (~5 kb) in the following amounts: 0.2, 0.5, 1.0, and 1.5 pg. Band intensities were measured with ImageJ and compared to band intensities from PCR products of genomic DNA preparations from 5 different mice. PCR was done twice for standard and sample DNA. For copy number estimations, the factor 10^6^ was used which reflects the size difference between the transgenic construct and the diploid mouse genome of ~ 5 Gb.

### Tissue preparation and fluorescence microscopy

Mice were killed by gassing with CO_2_ and the organs immediately removed. Fluorescence of organs was either visualized with an UV lamp (Fluotest Original Hanau, Hanau, Germany) at 254 nm wavelength and a custom digital camera, or under an inverse microscope (Keyence, Neu-Isenburg, Germany).

### Perfusion, slicing and immunohistochemistry

Mice were anesthetized with 70 mg/ml chloral hydrate (1 ml/100 g body weight) and perfused transcardially with phosphate-buffered saline (PBS) (130 mM NaCl, 7 mM Na_2_HPO_4_, 3 mM NaH_2_PO_4_, pH 7.4), followed by Zamboni´s solution (2 % paraformaldehyde, 15 % picric acid in 0.1 M phosphate buffer, pH 7.4). The brain was removed from the skull and incubated overnight in 30 % (w/v) sucrose/PBS. Coronal brainstem sections of 30 μm thickness were sliced using a microtome (MICROM GmbH, Walldorf, Germany), collected in 15 % (w/v) sucrose/PBS and washed twice in PBS and twice in PBST (PBS with 1 % (v/v) Triton X-100) (15 min each washing step). Sections were incubated for 1.5 h in blocking solution (2 % bovine serum albumin, 10 % goat serum and 0.3 % Triton X-100 in PBS, pH 7,4). Sections were transferred in blocking solution containing rabbit anti-GFP antibody (1:500, Invitrogen, Karlsruhe, Germany) and incubated overnight with agitation at 4 °C. After three washes with PBST (10 min each), sections were transferred into carrier solution (1 % bovine serum albumin, 1 % goat serum and 0.3 % Triton X-100 in PBS, pH 7,4) and treated with the secondary antibody anti-rabbit conjugated to Alexa Fluor 488 (1:500, Invitrogen). After 1.5 h with agitation at room temperature, slices were washed three times with PBS (10 min each) and mounted on glass slides with Vectashield Mounting Medium with DAPI (Vector Laboratories, Burlingame, USA). Photomicrographs were taken with an inverse microscope (Keyence) and the fluorescence was detected with the corresponding filter set.

### In vivo slice preparation and imaging

Mice were killed by gassing with CO_2_ and 150 μm thick living brainstem slices were cut with a vibratome (MICROM GmbH). During the preparation, slices were incubated in 95 % O_2_ and 5 % CO_2_ aerated ACSF solution (118 mM NaCl, 1 mM NaH_2_PO_4_, 25 mM NaHCO_3_, 3 mM KCl, 1 mM MgCl_2_, 1.5 mM CaCl_2_, pH 7.4). After preparation, brainstem slices were examined with a TCS SL confocal microscope (Leica, Nussloch, Germany) using 40-fold magnification and a PL FLUOTAR objective (40.0x/0.7 NA).

## Results and discussion

To generate a transgenic mouse line for in vivo cell labeling and the report of Cre-mediated recombination, we used the plasmid pCAGGS_cherry_Intron_GFP (Figure 1). This plasmid expresses the red fluorescent protein variant mCherry under the CAGGS promoter (chicken β-actin promoter with CMV enhancer), which is active in almost all tissues [22]. Upon Cre-mediated expression, mCherry is deleted and eGFP expressed.

### Pink colored mice

**Figure 2:**
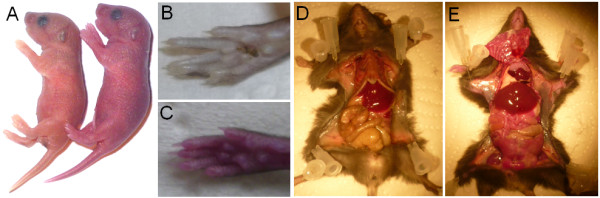
**The pink mouse. A**) A transgenic mCherry and its wild-type littermate are shown at postnatal day (P)0. B, C) Comparison of the paws of a young-adult wild-type (**B**) and a transgenic (**C**) mouse. The pink colored paw of the transgenic mouse is clearly visible without optical instruments. D, E) An adult wild-type mouse with an opened body (**D**) and its transgenic littermate (**E**), which displays reddish organs.

Pronuclear injection resulted in three founder mice. After crossing them back to wild-type mice, some of the young progeny of one founder had a pinkish skin (Figure 2A), which made them easily distinguishable from littermates. The pink skin color was still observed in the paw of adult animals (Figure 2B,C) and inside the body (Figure 2D,E) in the absence of UV light. These data indicated an extraordinarily high expression level of mCherry in this founder line. Genotyping PCR confirmed the presence of the reporter allele in these mice. The pink colored mice were fully viable and fertile, demonstrating, that the expression level of the mCherry protein had no toxic effects. This phenotype was observed both in the first and the second filial generation, indicating a generational persistence and no silencing of the transgene.

### Whole organ fluorescence

**Figure 3:**
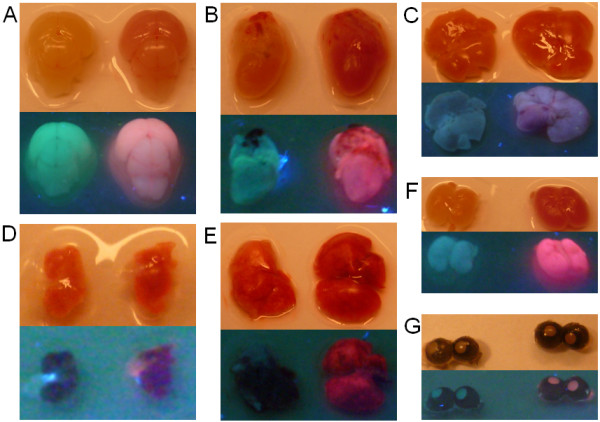
**Whole organ fluorescence under UV light.** Different organs of wild-type (left side) and transgenic (right side) P0 mice are shown. Upper panels represent bright field images and lower panels represent fluorescence images of different organs. **A**) brain; **B**) heart; **C**) liver; **D**) spleen; **E**) lung; **F**) kidney; and **G**) eyes. In contrast to the wild-type mouse, all organs of the transgenic mouse are reddish in the bright field and pink under UV light.

**Figure 4:**
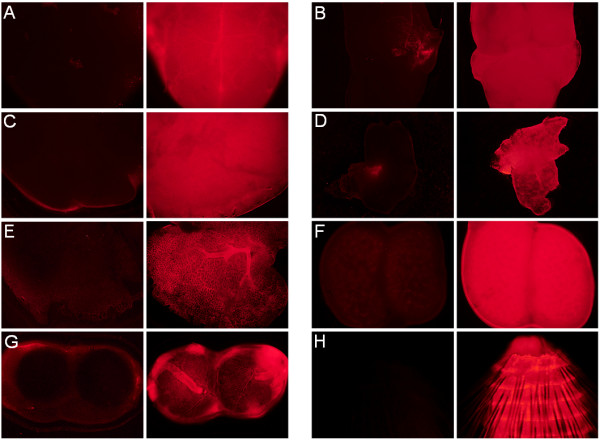
**Organ examination with the fluorescent microscope.** Organs of P0 mice were examined under a fluorescent microscope to show the difference in the fluorescence of wild-type (left side) and transgenic mice (right side). **A**) brain; **B**) heart; **C**) liver; **D**) spleen; **E**) lung, **F**) kidney, **G**) eye; and **H**) tail.

We next checked organs for fluorescence detection under UV light. The brain, heart, liver, lung, spleen, kidney, and eyes were taken from P0 mice and photographed in bright field and during excitation with UV light (Figure 3). Even without UV excitation, the organs of the transgenic mouse strain had a deeper shade of red than control littermates. After excitation with UV light, the difference between wild-type and transgenic mouse organs were also clearly visible. The organs of the mCherry mouse were pink in contrast to those of the littermates. In agreement with these results, all organs tested of the mCherry mouse line gave an intense shade of red under fluorescence microscopy (Figure 4). These data demonstrated high expression of mCherry in all of the organs analyzed.

### Copy number

**Figure 5:**
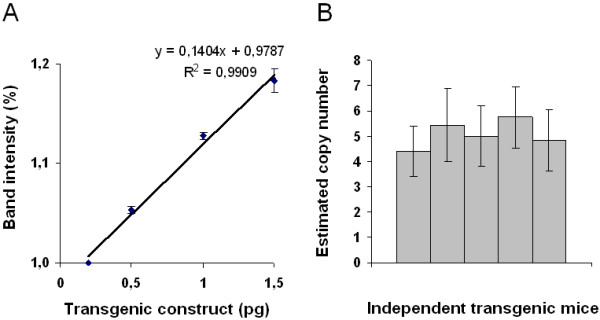
**Determination of transgene copy number by semi-quantitative RT-PCR.** Semi-quantitative RT-PCR experiments were performed on the initial construct (standard) or samples from mouse tail DNA preparations. **A**) Linear relation between template input (amount of the standard DNA) and measured band intensities of PCR products. **B**) Calculated copy numbers of five different transgenic mice. From each sample, the mean value and the standard deviation are shown. Experiments were performed in duplicate.

The high expression level of mCherry could result either from a high copy number of the transgene or from the integration site into the genome, thus representing a position effect. To estimate the copy number, we performed semi-quantitative PCR (Figure 5) on DNA from five different pink mice (Figure 5B). The data demonstrate ~ 4 to 6 copies per genome of a heterozygous mouse (5.1 ± 1.2 transgene copies in the haploid genome). This number indicates that the genomic area surrounding the integration site likely contributes to the high expression level of the transgene.

### Cre-mediated recombination

**Figure 6:**
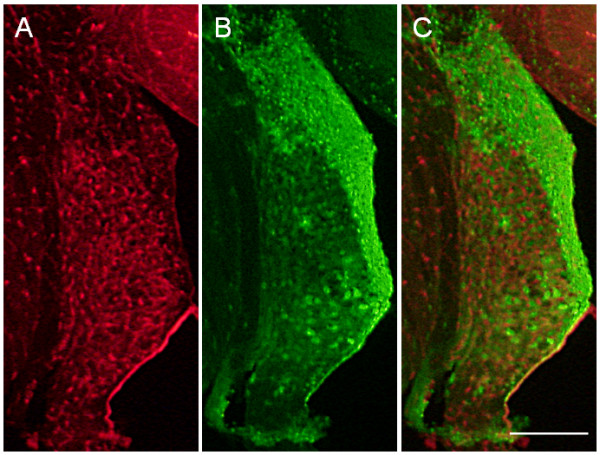
**Validation of Cre-mediated recombination.** The cochlear nucleus from a reporter mouse crossed with an *Egr2*^*Cre/+*^ driver line is shown after fixation. **A**) Red fluorescence, **B**) green fluorescence, **C**) merged picture. The strong green fluorescence demonstrates successful recombination. Scale bar is 200 μm.

Finally, we investigated the utility of the mCherry mouse as a Cre-reporter mouse line. Transgenic mice were therefore crossed with the *Egr2*^*Cre/+*^ Cre-driver mouse line [21], which expresses Cre in the auditory brainstem [23]. Fluorescent immunohistochemistry demonstrated expression of eGFP in the dorsal cochlear nucleus, a conspicuous structure of the auditory brainstem (Figure 6). A minority of cells expressed mCherry and eGFP in parallel, as indicated by their orange/yellow appearance (Figure 6C). This indicates incomplete recombination of all 4 to 6 copies, which is likely caused by low Cre expression in these cells. Despite this intermediate phenotype, these data demonstrate that our novel dual reporter mouse line is well suited to monitor Cre expression. The mixed expression of mCherry and eGFP might even be exploited to determine promoter activities, using Cre-driver lines.

**Figure 7:**
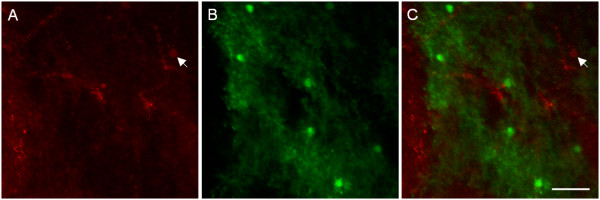
**In vivo imaging of brainstem slices.** Detection of non-recombined and recombined cells in a living brainstem slice from a reporter mouse crossed with an *Egr2*^*Cre*/+^ driver line. **A**) Red fluorescence, **B**) green fluorescence, **C**) merged picture. The green fluorescence demonstrates successful detection of recombined cells in vivo. Red cells represent non-recombined cells (arrow). Scale bar is 50 μm.

An important application of fluorescent proteins is their *in vivo* detection such as during electrophysiological characterization of neuronal subpopulations. We therefore analyzed whether red and green fluorescence labeled cells can be detected *in vivo*. Indeed, both non-recombined (red) and recombined (green) cells were easily recognized in unfixed, living brain slices (Figure 7). These data demonstrate the usefulness of our reporter mouse for in vivo monitoring and detection of recombination.

## Conclusions

This novel pink Cre-reporter mouse line represents a versatile tool in transgenic mouse research. A great advantage is the high expression level of the fluorescent protein mCherry, which allows easy recognition and separation of animals harboring the reporter allele from littermates without further analysis. The presence of two fluorescent proteins is useful for parallel in vivo monitoring of recombined and non-recombined cells, *in vivo* recordings, and analyses of cellular connectivity. Finally, this mouse line is well suited for teaching purposes. Its pink color can be used to easily demonstrate the power of transgenic technologies and crossed with an ubiquitously expressing Cre-driver, the color would disappear.

## Competing interests

The authors declare that they have no competing interests.

## Authors’ contributions

HH and SVS carried out the experiments. HH drafted and wrote the manuscript with the help of HGN. All authors read and approved the final manuscript.
